# Deep vein thrombosis in a patient of adenomatous polyposis coli treated successfully with aspirin: A case report

**Published:** 2016

**Authors:** Neha Agrawal, Tuhin Santra, Arnab Kar, Pradipta Guha, Mita Bar, Apu Adhikary, Sumana Datta

**Affiliations:** 1 Department of Medicine, Calcutta National Medical College and Hospital, Kolkata, India.; 2 Department of Medicine, Gangarampur Subdivisional Hospital, India.; 3 Department of Medicine, North Bengal Medical College & Hospital, India.; 4 Department of Family Medicine, Calcutta Medical Research Institute, Kolkata, India.

**Keywords:** Deep vein thrombosis, Adenomatous polyposis coli, Diarrhea, Aspirin

## Abstract

**Background:**

Deep vein thrombosis is an important cause of morbidity and mortality. However, its association with adenomatous polyposis coli is extremely rare. Here we present an interesting case of deep vein thrombosis associated with adenomatous polyposis coli.

**Case Presentation:**

A 15 year old female who was having fever and diarrhea for 5 months developed bilateral asymmetric painful swelling of lower limbs for 1 month. Doppler ultrasound of lower limbs revealed presence of thrombosis from inferior vena cava up to popliteal vein. Colonoscopy and biopsy were suggestive of adenomatous polyposis coli. However, she could not tolerate anticoagulant therapy and was put on aspirin therapy for 6 months to which she responded well with the resolution of thrombus.

**Conclusion:**

Role of aspirin therapy may be considered whenever a patient of venous thrombosis cannot tolerate anticoagulant therapy.

**V**enous thromboembolism is an important cause of hospital acquired morbidity and mortality (1). Its association with adenomatous polyposis coli is a rare event (2, 3). Malignancy or major operative procedure associated with colonic polyp may predispose to thromboembolic event (2). There has been previously published few reports of diarrhea associated with deep vein thrombosis (4-6). Etiology of diarrhea in those cases was unrelated to colonic polyp. However, association of deep vein thrombosis with non-malignant colonic polyp presenting with diarrhea is an extremely rare event with a report of only one case in previous English literature (3). Mainstay of therapy of deep vein thrombosis is anticoagulation. However, fatal bleeding episode following anticoagulation in our patient lead to withdrawal of anticoagulation therapy. The patient was treated successfully with low dose aspirin therapy (75mg/day) with complete resolution of thrombus. 

## Case Presentation

A 15-year-old unmarried female presented to us with complain of asymmetric onset lower limb swelling for last 1 month. The swelling initially involved the left lower limb and for last 1 week she had also developed right lower limb swelling. The swelling which was initially painless later became painful. 

She was also having low grade irregular fever for last 5 months along with small amount of non-foul smelling mucus containing recurrent episodes of diarrhea for the same duration along with a history of significant weight loss. She had an episode of hematochezia 1 day after admission. There was no history of abdominal pain, vomiting, abdominal distension, facial puffiness, oliguria, bleeding from any other sites, joint pain, skin rash, recent contact with tuberculosis, recent surgery or prolonged immobilization. She had mild pallor and her body mass index (BMI) was 17.5 kg/mt^2^. She had bilateral pitting pedal edema along with raised local temperature and a positive Homans’ sign and Moses’ sign.

**Figure 1:**
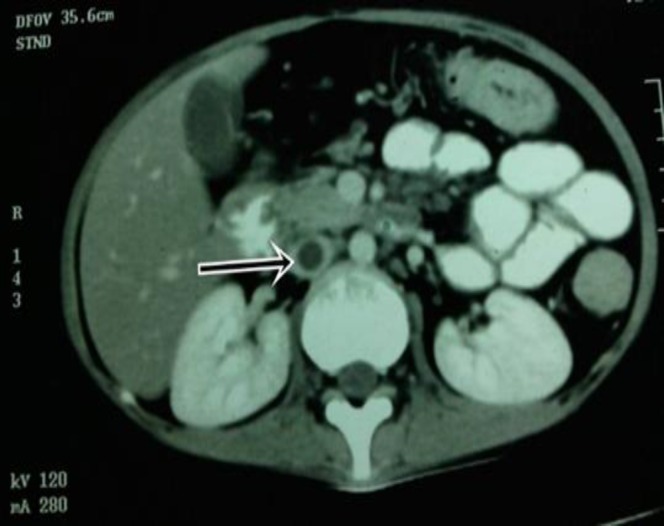
CT abdomen showing thrombus in IVC

Her routine investigations revealed a hemoglobin level of 8.9 g/dl, total leucocyte count (TLC) of 10,500/mm^3^, erythrocyte sedimentation rate (ESR) of 6 mm. in 1^st^ hour, low albumin level (2.5g/dl) and serum creatinine of 0.9 mg/dl. Stool examination revealed presence of mucous, blood and pus cells. Chest x-ray, ultrasonography of whole abdomen was unremarkable. She was found to be HIV negative. Duplex color doppler of both lower limbs revealed presence of intraluminal thrombus involving the inferior vena cava (IVC) from the level just beyond the origin of left renal vein up to the popliteal vein. Contrast enhanced CT (computed tomography) of abdomen was done which confirmed the presence of IVC (inferior vena cava) thrombosis (figure 1). 

**Figure 2:**
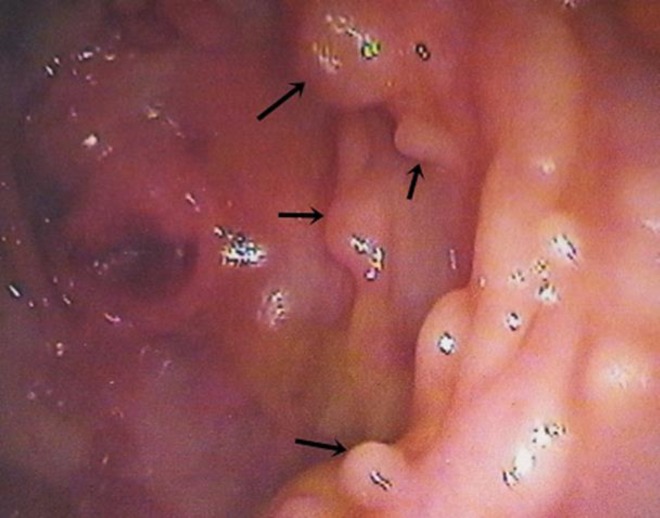
Multiple polyps in colon

**Figure 3:**
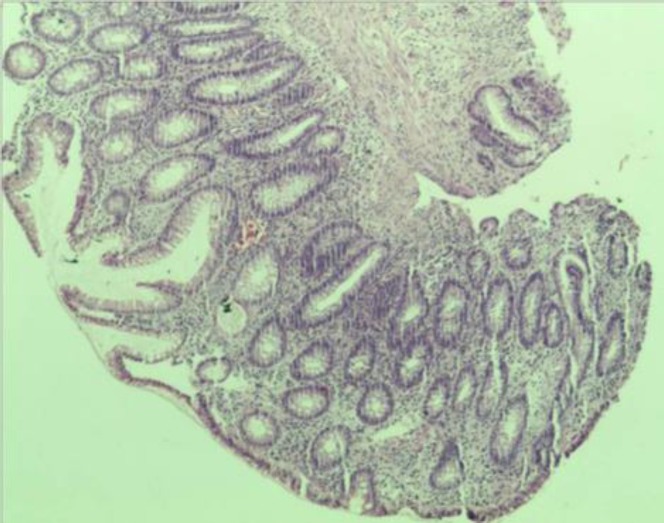
Biopsy from sigmoid polyp revealing adenomatous polyp without any evidence of malignancy

Colonoscopy revealed presence of multiple polyps, may be up to 100 in number with size being 0.5-1 cm. of diameter up to the level of cecum (Figure 2). Biopsy from the sigmoid polyp revealed adenomatous polyp without any evidence of malignancy (figure3). Her prothrombin time (P time), activated partial thromboplastin time (aPTT) and fibrinogen level was within normal limit and she had a raised fibrin degradation product (FDP) level (2671.2 ng/ml). Her antinuclear antibody (ANA), anti-double stranded DNA (anti ds-DNA), lupus anticoagulant and anticardiolipin antibody was negative and she had a normal homocysteine and protein C and protein S level.

So, she was diagnosed to have adenomatous polyp with deep vein thrombosis and she was put on injection enoxaparin and tablet warfarin. But 2 days after she developed life threatening hematochezia and patient went to shock and the therapy was stopped. Repeat P time was normal. The patient was successfully resuscitated and after hematochezia was stopped, she was put on tablet warfarin but again she developed hematochezia. Thus, both the enoxaparin and warfarin therapy were discontinued. After control of hematochezia she was then put on a trial of aspirin therapy (75 mg/day). No bleeding manifestations occurred and repeat doppler ultrasound study after 2 months revealed partial recanalization within the thrombus with resolution of her symptoms. Doppler ultrasound after 4 months revealed complete recanalization and aspirin therapy was continued for a total duration of 6 months.

## Discussion

Venous thromboembolism is associated with several risk factors. Prolonged immobility, major surgery, malignancy, trauma, presence of antiphospholipid antibody, use of oral contraceptives are important leading causes of thromboembolic event (7, 8). 

It may occur in association with other chronic inflammatory conditions like inflammatory bowel disease, tuberculosis or with malabsorption syndromes which present with abdominal symptoms like diarrhea, pain abdomen, ascites (5, 6, 9). However, to the best of our knowledge, deep vein thrombosis associated with non-malignant colonic polyp has been described only once in previous English literature (3). 

In that case, deep vein thrombosis was postulated secondary to immobility associated with poor health which is not applicable to our case. Dehydration associated with diarrhea might be an explainable risk factor for development of deep vein thrombosis in our patient (10).

Again, major bleeding episode associated with anticoagulant therapy prevented its further use in our patient. Aspirin has been tried as thromboprophylaxis for venous thromboembolism in high risk patients (11, 12). However, its use for venous thrombosis is still not recommended although it is recommended for arterial thromboembolic event. Aspirin which suppresses cell proliferation by inhibiting prostaglandin synthesis has been assessed as possible inhibitors of colon cancer (13). We treated our patient successfully with aspirin 75 mg/day for 6 months with complete resolution of symptoms and thrombus.

## Conclusion

This is a well-documented clinical, radiological and histological case report of recognized association of deep vein thrombosis with adenomatous polyposis coli. Role of aspirin therapy may be considered whenever a patient of venous thrombosis cannot tolerate anticoagulant therapy.
